# Synthesis and Reactions of α-Hydroxyphosphonates

**DOI:** 10.3390/molecules23061493

**Published:** 2018-06-20

**Authors:** Zita Rádai, György Keglevich

**Affiliations:** Department of Organic Chemistry and Technology, Budapest University of Technology and Economics, 1521 Budapest, Hungary; radai.zita@mail.bme.hu

**Keywords:** α-hydroxyphosphonate, Pudovik reaction, *O*-derivatization, oxidation, substitution, rearrangement, hydrolysis

## Abstract

This review summarizes the main synthetic routes towards α-hydroxyphosphonates that are known as enzyme inhibitors, herbicides and antioxidants, moreover, a number of representatives express antibacterial or antifungal effect. Special attention is devoted to green chemical aspects. α-Hydroxyphosphonates are also versatile intermediates for other valuable derivatives. *O*-Alkylation and *O*-acylation are typical reactions to afford α-alkoxy-, or α-acyloxyphosphonates, respectively. The oxidation of hydroxyphosphonates leads to ketophosphonates. The hydroxy function at the α carbon atom of hydroxyphosphonates may be replaced by a halogen atom. α-Aminophosphonates formed in the nucleophilic substitution reaction of α-hydroxyphosphonates with primary or secondary amines are also potentially bioactive compounds. Another typical reaction is the base-catalyzed rearrangement of α-hydroxy-phosphonates to phosphates. Hydrolysis of the ester function of hydroxyphosphonates leads to the corresponding phosphonic acids.

## 1. Synthetic Routes towards α-Hydroxyphosphonates

**Scheme 1:**
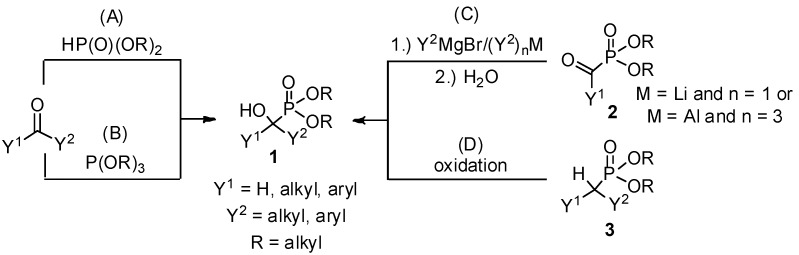
Synthetic pathways towards α-hydroxyphosphonates **1**.

Due to the bioactivity of α-hydroxyphosphonates **1**, the synthesis of these derivatives is an evergreen topic in organophosphorus chemistry [1,2,3,4,5,6,7,8]. The main approaches to obtain α-hydroxyphosphonates **1** are shown in Scheme 1. The most commonly studied method is the addition of a dialkyl phosphite to an oxo compound (method “A”) [9]. The good atom economy makes this method the most appealing way to synthesize α-hydroxyphosphonates **1**. In the majority of cases, the addition is carried out in the presence of a base catalyst, but a few acid-catalyzed variations are also known. An alternative route is the condensation of an oxo compound and a trialkyl phosphite (method “B”) [10]. In contrast to method “A”, this reaction is usually catalyzed by different acids. In the literature, the denomination of the reactions is not consistent, as both methods “A” and “B” are referred to as the Pudovik and Abramov reaction, and also as the phospha-aldol reaction. The third main approach towards α-hydroxyphosphonates **1** involves the reaction of α-ketophosphonates **2** with Grignard reagents [11] or other organometallic compounds [11,12], followed by hydrolysis (method “C”). α-Alkyl phosphonates **3** may also be converted to the corresponding α-hydroxy derivatives by oxidation (method “D”) [13,14]. In this review, methods “A” and “B” are discussed in detail. Although the asymmetric synthesis of α-hydroxyphosphonates **1** is of special importance [4,5,6,7,8], in this review the discussion is limited to racemic derivatives.

### 1.1. Synthesis of α-Hydroxyphosphonates by the Reaction of Aldehydes/Ketones and Dialkyl Phosphites

The reaction of oxo compounds and dialkyl phosphites catalyzed by alkali alcoholates (Scheme 1, method “A”) was first reported by Pudovik [15]. Since then, a number of variants involving different catalysts and conditions were elaborated to obtain α-hydroxyphosphonates by the reaction under discussion (Table 1). Recent methods have targeted the use of inexpensive and simple catalysts, and mild reaction conditions in the spirit of green chemistry. In the great majority of the cases, the addition was carried out without using any solvent. It is worth mentioning that starting from ketones, the accomplishment of the reaction is more challenging than in the cases applying aldehydes. The synthesis of α-alkyl-α-hydroxyphosphonates often requires the use of “exotic” catalysts [16,17,18,19,20], or an excess of a base. Procedures also applicable for the conversion of ketones are marked by an asterisk in Table 1.

A base-catalyzed variation of the Pudovik reaction was carried out in the presence of 5 mol % potassium phosphate as the catalyst (Table 1/Entry 1) [21]. This method has the advantage of using an inexpensive catalyst, requiring short reaction times and an easy work-up procedure that comprises simple extraction, and, in most cases, provides the products in high yields. Barium hydroxide was also an efficient catalyst in the addition [22,23]. One method employed 10 mol % Ba(OH)_2_ (Table 1/Entry 2) [22], while according to another protocol, Ba(OH)_2_∙8H_2_O was used (Table 1/Entry 3) [23]. The first Ba(OH)_2_-catalyzed variation suffers from the drawback of a tedious work-up procedure involving extraction, and washing, followed by crystallization [22], while in the latter case, the use of THF as the solvent is a disadvantage [23]. One equivalent of magnesium oxide efficiently catalyzed the addition of diethyl phosphite to substituted benzaldehydes (Table 1/Entry 4) [24]. The method could be extended to the conversion of γ-cyanoketones by applying the base in a twofold quantity (Table 1/Entry 5) [25]. The reaction of dimethyl phosphite with oxo compounds including ketones afforded the corresponding α-hydroxyphosphonates **1** in the presence of three equivalents of aluminum oxide after stirring for 72 h (Table 1/Entry 6) [26]. The reaction time could be shortened dramatically by using potassium fluoride with only one equivalent of Al_2_O_3_. However, this latter method was efficient only in case of aldehydes as the starting compounds (Table 1/Entry 7) [27]. The addition was also performed in the presence of one equivalent of triethylamine as the catalyst (Table 1/Entry 8) [28]. A simple crystallization afforded the desired products in good to excellent yields. Another method employed three equivalents of triethylamine together with one equivalent of magnesium chloride (Table 1/Entry 9) [29]. In the latter case, an extraction was inserted before the crystallization step in the work-up procedure [29]. In agreement with the requirements of environmentally friendly approaches, green activation methods, such as microwave (MW) irradiation [30,31] and grinding [32,33] were also employed in the synthesis of α-hydroxyphosphonates **1**. According to a MW-assisted procedure, the reaction of benzaldehydes and diethyl phosphite was carried out without the use of any catalyst or solvent (Table 1/Entry 10) [30], while another method combined MW irradiation with sodium carbonate as the base (Table 1/Entry 11) [31]. Na_2_CO_3_ was also used efficiently in another protocol, when the contact among the reaction components was promoted by grinding (Table 1/Entry 12) [32]. The drawback of the method is the complicated work-up procedure comprising washing, extraction, and finally recrystallization. A similar protocol utilizes the piperazine-catalyzed reaction of aldehydes and diethyl phosphite in a mill (Table 1/Entry 13) [33]. In the latter case, the work-up involved washing, extraction and column chromatography. The synthesis of α-hydroxyphosphonates **1** was also carried out in the presence of 10 mol % choline hydroxide as an IL catalyst (Table 1/Entry 14) [34]. According to a plausible mechanism suggested by the authors, the IL promotes the reaction by activation through *H*-bridges. An interesting protocol is a special fluoroapatite-catalyzed Pudovik reaction (Table 1/Entry 15) [35]. A mixture of the starting dialkyl phosphite and oxo compound was stirred with a spatula, and then left standing on the catalyst over 1–1.5 min. The work-up involved extraction with dichloromethane, and recrystallization of the crude product. Acid-catalyzed variations of the Pudovik reaction occur rarely in the literature [36,37]. Potassium hydrogensulfate proved to be an efficient catalyst in an amount of 20 mol % in addition of diethyl phosphite to substituted aldehydes (Table 1/Entry 16) [36]. According to another method, silica-supported tungstic acid was applied as the catalyst (Table 1/Entry 17) [37]. Organometallic compounds may also play the role of the catalyst during the synthesis of α-hydroxyphosphonates **1** [38,39,40]. Butyllithium was applied in the amount of 0.1 mol % in hexane as the solvent (Table 1/Entry 18) [38]. The method not only bears the disadvantages of using an exotic catalyst in an inert atmosphere, but also the need for a complicated work-up involving quenching, washing, and finally column chromatography. As for the advantages, mild reaction conditions and wide substrate scope including ketones can be mentioned. Titanium tetraisopropylate was also applied to catalyze the reaction of ketones and dimethyl phosphite (Table 1/Entry 19) [39]. Column chromatography of the crude product afforded α-alkyl α-hydroxyphosphonates **1** in good to excellent yields. Molybdenum dioxide dichloride (MoO_2_Cl_2_) was also tested as the catalyst in the Pudovik reaction (Table 1/Entry 20) [40]. Using it in an amount of 5 mol % under solvent-free conditions at 80 °C, the hydroxyphosphonates **1** were obtained good yields.

### 1.2. Synthesis of α-Hydroxyphosphonates by the Reaction of Aldehydes/Ketones and Trialkyl Phosphites

Besides the reaction of oxo compounds with dialkyl phosphites discussed in Section 1.1 (Scheme 1, method “A”), the other widespread method for the synthesis of α-hydroxyphosphonates is the condensation of aldehydes or ketones with trialkyl phosphites (Scheme 1, method “B”) [10]. This way is of somewhat less importance due to the reduced atom economy, as compared to the major route outlined. While the reaction involving dialkyl phosphites is usually catalyzed by different bases, the typical catalysts for the condensation of oxo compounds with trialkyl phosphites exhibit acidic character (Table 2).

A method of choice is a solvent- and catalyst-free accomplishment under ultrasonic irradiation (Table 2/Entry 1) [41]. The reaction of aldehydes and trialkyl phosphites at 25 °C was complete after 10–35 min and provided the α-hydroxyphosphonates **1** in excellent yields after crystallization (Table 2/Entry 1). The method was successfully applied to ketones as starting materials as well. Another ultrasound-assisted variation is the potassium dihydrogen phosphate-catalyzed reaction (Table 2/Entry 2) [42]. According to a plausible mechanism, the role of the acid catalyst is the protonation of the carbonyl function of the aldehyde making the C=O bond more electrophilic, and hence facilitating the nucleophilic attack of the (RO)_3_P reagent. A similar procedure employed sulphamic acid as the catalyst (Table 2/Entry 3) [43]. Again, the catalytic effect of the sulphamic acid was attributed to its capability to protonate the oxo compound. The camphorsulfonic acid-catalyzed syntheses of α-hydroxyphosphonates **1** from aldehydes and triethyl phosphite was carried out under ultrasonic irradiation on stirring (Table 2/Entry 4) [44]. Applying the ultrasound technique, the reaction times could be shortened from 30–75 min to 8–15 min after comparing the results with those obtained without ultrasonication. At the same time, the yields were similar (85–93%) for the two procedures [44]. Oxalic acid also proved to be an efficient catalyst in the condensation under discussion at 80 °C (Table 2/Entry 5) [45]. A series of organic solvents were tested as the reaction medium, but the desired products were obtained in low yields (<30%). Under neat conditions, the α-hydroxyphosphonates **1** were formed efficiently as suggested by the yields of 83–98% obtained after quenching, extraction and column chromatography. In the hope of synthesizing a new class of antibiotics, α-hydroxy-phosphonates **1** containing a β-lactam scaffold were prepared in the presence of tartaric acid as the catalyst (Table 2/Entry 6) [46]. 

Among a series of acids (e.g., acetic acid, trifluoroacetic acid, *p*-toluenesulfonic acid, lactic acid, fumaric acid and tartaric acid) screened as potential catalysts of the reaction, fumaric acid and tartaric acid were found to be the most efficient. It was concluded that weak acids (with a pKa value around 4.5) are the best catalysts of the reaction [46]. According to another procedure, pyridine-2,6-dicarboxylic acid was applied as the catalyst (Table 2/Entry 7) [47]. The reaction of oxo compounds including ketones with trimethyl phosphite was carried out in water at 50 °C to furnish hydroxyphosphonates. In another approach, the pyridine-based catalyst was replaced by guanidine hydrochloride (Table 2/Entry 8) [48]. The drawback of this protocol is the rather complicated work-up comprising quenching, extraction, and then chromatography. An interesting finding is that iodine may also be a catalyst in the reaction of aldehydes and triethyl phosphite (Table 2/Entry 9) [49]. The catalytic effect of I_2_ was explained similarly as that of the acid catalysts: iodine is able to interact with the oxygen atom of the carbonyl group, making the C=O bond more electrophilic. β-Cyclodextrin was found to be a promising and recoverable catalyst of the condensation (Table 2/Entry 10) [50]. However, it was necessary to use this catalyst in a one equivalent quantity, and completion of the reaction required longer times (8–12 h) as compared with other protocols. Ammonium metavanadate was tested as the catalyst in the reaction of benzaldehydes with triethyl phosphite, as well as with diethyl phosphite [51]. In the first case, the reaction was complete after 12–25 min (Table 2/Entry 11). However, in the second case, the desired products were only formed in traces. Metal salts were also applied in the reaction of oxo compounds and trialkyl phosphites with success (Table 2/Entries 12–14) [52,53,54]. The reaction of slightly electron-rich benzaldehydes and triethyl phosphite in the presence of ZnBr_2_ afforded the corresponding α-hydroxyphosphonates (Table 2/Entry 12) [52]. Bismuth(III) nitrate pentahydrate was applied efficiently as the catalyst in the reaction of aldehydes and triethyl phosphite under MW irradiation at 70 °C (Table 2/Entry 13) [53], while the niobium(V)chloride-trimethylsilyl chloride system was used in an amount of only 0.05 mol % at 25 °C to prepare hydroxyphosphonates (Table 2/Entry 14) [54].

### 1.3. The “Greenest” Protocol for the Synthesis of α-Hydroxyphosphonates

As it was presented in Section 1.1 and Section 1.2, a great number of procedures have been elaborated for the synthesis of α-hydroxyphosphonates **1** from oxo compounds by reaction with dialkyl or trialkyl phosphites. Although, several methods involved the use of inexpensive and easily available catalysts together with mild and solvent-free reaction conditions, the work-up procedures still suffered from drawbacks. In most cases, the crude product was extracted with organic solvents, and then purified by column chromatography or recrystallization. The combination of the purification steps mentioned is also a frequent option [22,32,33,35,38,48].

**Scheme 2:**
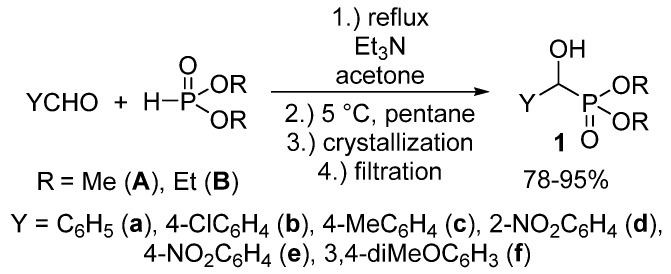
A green, solvent-economical synthetic method towards α-hydroxyphosphonates (**1**).

We aimed at the elaboration of a new, environmentally-friendly, procedure for the synthesis of α-hydroxyphosphonates **1**. As opposed to the procedures already published, we wished to reduce the amount of volatile organic solvents used not only during the reaction, but also in the course of the work-up procedure. According to our method, an equimolar mixture of substituted benzaldehyde and dialkyl phosphite was stirred at reflux in the presence of 10 mol % of triethylamine as the catalyst, in a minimal amount of acetone (1.0 mL/11.0 mmol of the reagents). Then, *n*-pentane was added to the mixture, which was then cooled to 5 °C. The desired product crystallized from the reaction mixture, and the α-hydroxyphosphonates **1** could be obtained easily by a simple filtration in good to excellent yields (78–99%) in a pure form. The main novelty of our protocol is the absence of further purification steps, a consequence of the “one-pot” synthesis and precipitation from the reaction mixture (Scheme 2) [55].

**Scheme 3:**
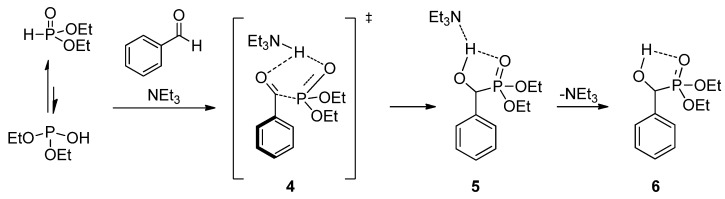
Calculated mechanism of the triethylamine-catalyzed Pudovik reaction.

To reveal the role of triethylamine in the addition, the reaction of benzaldehyde with diethyl phosphite was investigated by quantum chemical calculations [56]. It was found that in the absence of a catalyst, the enthalpy of activation is around 85.9 kJ/mol, and the addition is thermoneutral. The calculations revealed that triethylamine is able to promote the proton transfer from the >P(O)H reagent to the oxo compound. The amine-catalyzed reaction goes with a decreased enthalpy of activation of 68.8 kJ/mol, and follows an exothermic route (∆H = −17.2 kJ/mol). The suggested mechanism involving transition state **4** and intermediate **5** is shown in Scheme 3.

In conclusion, we were successful in the elaboration of another green protocol for the synthesis of α-hydroxyphosphonates **1** from substituted benzaldehydes and a series of dialkyl phosphites in the presence of triethylamine as the catalyst [55]. The new method involves the preparation in boiling acetone followed by crystallization of the product in high purity. There was no need for additional purification. The role of triethylamine in the reaction was justified by quantum chemical calculations [56].

## 2. Reactions of α-Hydroxyphosphonates

**Scheme 4:**
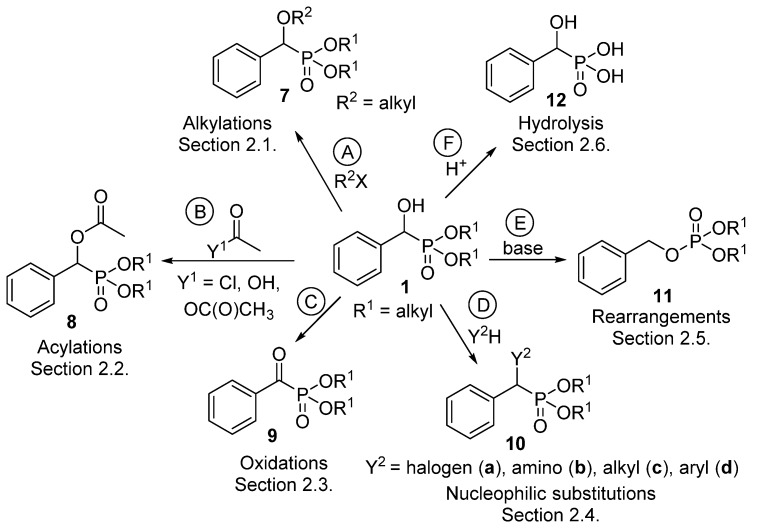
Family tree of compounds **7**–**12** derived from α-hydroxyphosphonates (**1**).

α-Hydroxyphosphonates **1** deserve interest not only as potential enzyme inhibitors [57], herbicides [58], antibiotics [59] and antibacterial or antifungal [60] agents, but also as starting materials for other valuable derivatives [61]. Section 2.1 summarizes the alkylation reactions of α-hydroxyphosphonates (Scheme 4, Route “A”). Acylation of the hydroxy function is one of the most extensively studied transformations within this family of compounds (Scheme 4, Route “B”, Section 2.2). A series of α-acyloxyphosphonates (**8**) was synthesized applying different acylating agents, like carboxylic and sulfonyl chlorides, as well as carboxylic anhydrides and acids. The oxidation of α-hydroxyphosphonates leads to α-ketophosphonates **9** (Scheme 4, Route “C”, Section 2.3). Another thoroughly studied field is nucleophilic substitution at the α carbon atom of α-hydroxyphosphonates to afford α-halo-, and α-amino or α-alkylphosphonates **10** (Scheme 4, Route “D”, Section 2.4). The rearrangement of hydroxyphosphonates to benzyl phosphates **11** is an interesting field (Scheme 4, Route “E”, Section 2.5). The curiosity of this transformation is the high substrate-dependence. Last but not least, α-hydroxyphosphonic acids **12** may be obtained by the hydrolysis of the ester function of α-hydroxyphosphonates (Scheme 4, Route “F”, Section 2.6).

The derivatives **7**–**12** synthesized from α-hydroxyphosphonates were summarized in the family tree shown in Scheme 4. Classes **7**–**12** represent potentially bioactive compounds.

### 2.1. Alkylations

**Scheme 5:**
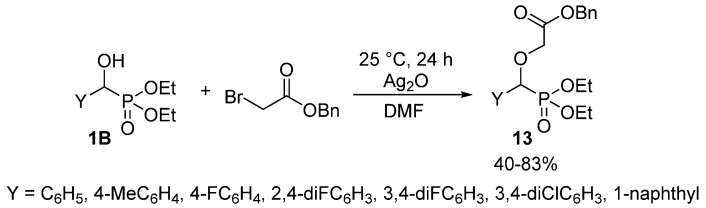
*O*-alkylation of hydroxyphosphonates **1B** with benzyl bromoacetate.

Alkylation of the hydroxy function of α-hydroxyphosphonates **1** does not belong to their extensively studied reactions, as only a few articles reported the synthesis of α-alkoxyphosphonates **7**. A related article targeted antimalarial drugs, and the synthesis involved the *O*-alkylation of hydroxyphosphonates **1B** with benzyl bromoacetate in the presence of silver oxide (Scheme 5) [62].

**Scheme 6:**
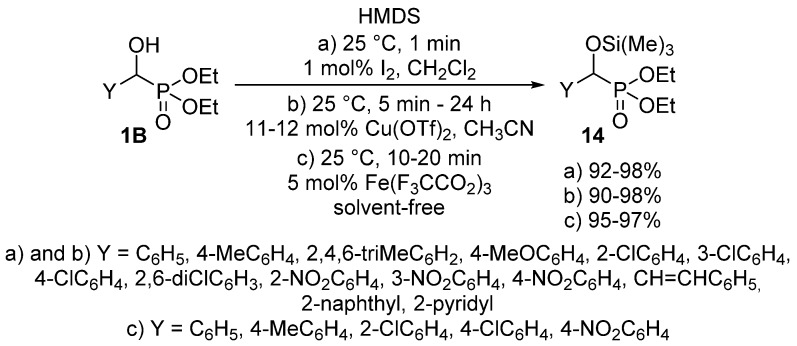
Possible ways of introducing a silyl protecting group to α-hydroxyphosphonates **1B**.

The protection of the α-hydroxy function may also occur in the literature as another utilization of *O*-alkylation. A tetrahydropyranyl (THP) protecting group was introduced to an α-hydroxyphosphonate **1B** by treatment with 3,4-dihydro-2*H*-pyran applying *p*-toluenesulfonic acid as the catalyst [63]. The silylation of α-hydroxyphosphonates **1** is also a useful tool to protect the hydroxy function [64,65,66,67]. The reaction of hydroxyphosphonates **1B** with hexamethyldisilazane (HMDS) was studied using a great variety of catalysts including iodine (Scheme 6, Route “a”) [65], copper triflate (Scheme 6, Route “b”) [66] or iron(III) trifluoroacetate (Scheme 6, Route “c”) [67].

**Scheme 7:**
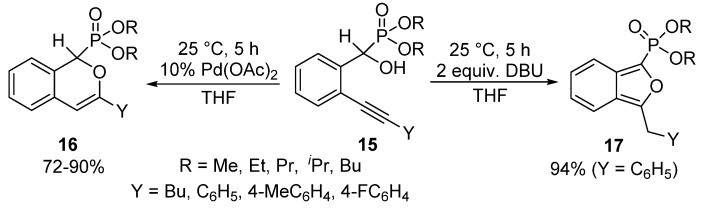
Selective cyclization of *ortho*-alkynyl α-hydroxyphosphonates **15**.

A unique protocol has been reported for the cyclization of α-hydroxyphosphonates bearing an alkynyl function in the *ortho*-position of the aromatic ring (**15**) [68]. Applying Pd(II) acetate as the catalyst, the nucleophilic attack of the α-*O* atom occurred on the more distant *C*-atom of the triple bond of the alkynyl substituent to form the six-membered product **16** selectively. Surprisingly, changing the Pd(OAc)_2_ catalyst to DBU used in 2 equivalents led to a five-membered cyclic product **17**. In the latter case, the attack of the hydroxy group occurred on the nearer *C*-atom of the triple bond, followed by isomerization (Scheme 7).

### 2.2. Acylations

The synthesis of α-acyloxyphosphonates **8** is one of the most thoroughly studied transformations of α-hydroxyphosphonates **1**. α-Acyloxyphosphonates **8** attracted attention as potential herbicides [69,70,71], fungicides [69], and insecticides [72]. A number of articles reported the acylation of α-hydroxyphosphonates **1** with carboxylic acid chlorides in the presence of triethylamine [73,74,75] or pyridine [76,77] as the hydrochloric acid scavenger. Among the –C(O)Cl reagents, phenoxyacetyl chloride derivatives **18** are frequently applied acylating agents [70,71,76,77,78,79,80,81,82,83,84]. 

**Scheme 8:**
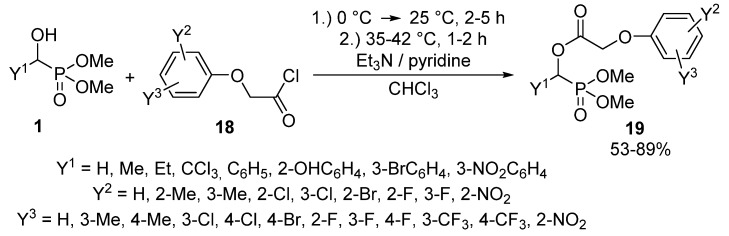
Synthesis of α-acyloxyphosphonates (**19**) from α-hydroxyphosphonates (**1**).

In general, the reaction of α-hydroxyphosphonates **1** with phenoxyacetyl chlorides **18** required a reaction time of 3–7 h, and the corresponding α-acyloxyphosphonates **19** were isolated in yields of 53–89% (Scheme 8) [78]. The compounds **19** thus-obtained are popular target molecules, as a series of related derivatives exhibited herbicidal activity [70,76,77,78,79,80,83,84]. 

**Scheme 9:**
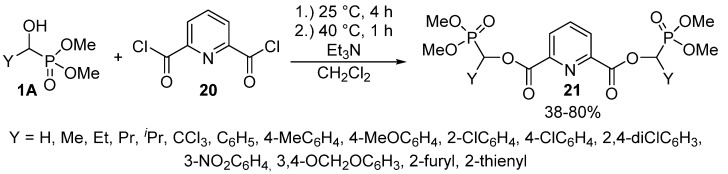
Acylation of α-hydroxyphosphonates (**1A**) with 2,6-pyridinedicarboxylic acid chloride (**20**).

The reaction of α-hydroxy-phosphonates **1A** with 2,6-pyridinedicarboxylic acid chloride (**20**) led to bis[dimethyl phosphonomethyl pyridine-2,6-dicarboxylates] **21** containing two α-acyloxyphosphonate moieties (Scheme 9). The reaction took place under mild conditions, and the products **21** were isolated with variable yields (38–80%). The derivatives so-obtained **21** also showed herbicidal activity [85].

**Scheme 10:**
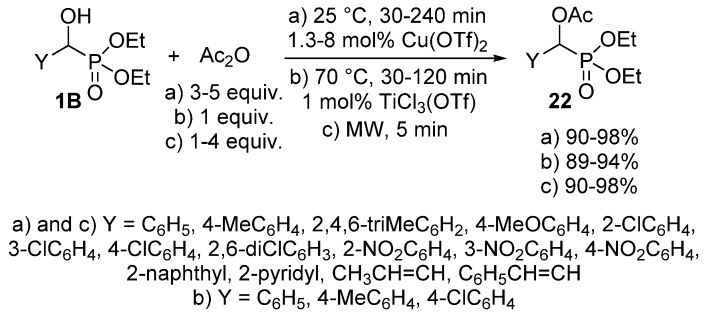
Possible ways for the synthesis of α-acetyloxyphosphonates **22**.

The acylation of α-hydroxyphosphonates **1B** has been also performed with acetic anhydride [86,87,88]. This reaction was carried out in the presence of a catalytic amount (1.3–8 mol %) of Cu(OTf)_2_ at 25 °C to afford the desired α-acetyloxyphosphonates **22** almost quantitatively (Scheme 10, Route “a”) [86]. In another instance, 1 mol % of TiCl_3_(OTf) was employed as the catalyst (Scheme 10, Route “b”). In this case, completion of the reaction required a somewhat higher temperature (70 °C) [87]. A more benign synthetic route to α-acetyloxyphosphonates **22** is the reaction of α-hydroxyphosphonates (**1B**) and acetic anhydride under MW irradiation (Scheme 10, Route “c”) [88]. The acylation was complete after a short reaction time of 5 min, and the products were obtained in excellent yields (90–98%). However, a lack of control of the reaction temperatures is a shortcoming that prevents reproduction.

The synthesis of α-acyloxyphosphonates **8** was also carried out starting from substituted benzaldehydes, dimethyl phosphite and acetic anhydride in the presence of magnetic nanoparticle-supported guanidine [89]. In this one-pot procedure, the formation of the α-hydroxyphosphonate **1** in the Pudovik reaction was followed by acylation of the hydroxy function.

**Scheme 11:**
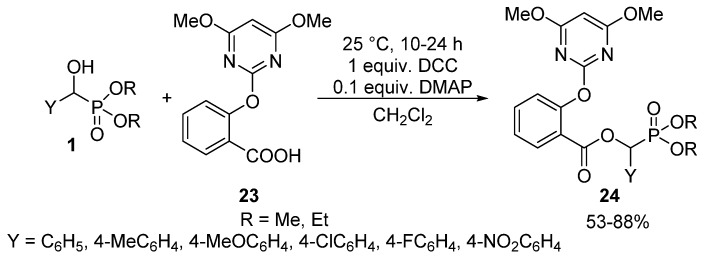
Coupling reaction of α-hydroxyphosphonates (**1**) with carboxylic acid **23**.

Carboxylic acids may also be efficient acylating agents of α-hydroxyphosphonates **1** in the presence of *N*,*N*′-dicyclohexylcarbodiimide (DCC) as the coupling reagent, and 4-dimethyl-aminopyridine (DMAP) as the base [27,90,91]. This is exemplified by the reaction of 2-(4,6-dimethoxypyrimidin-2-yloxy)benzoic acid (**23**) at 25 °C to give α-acyloxyphosphonates **24** possessing herbicidal activity (Scheme 11) [27].

**Scheme 12:**
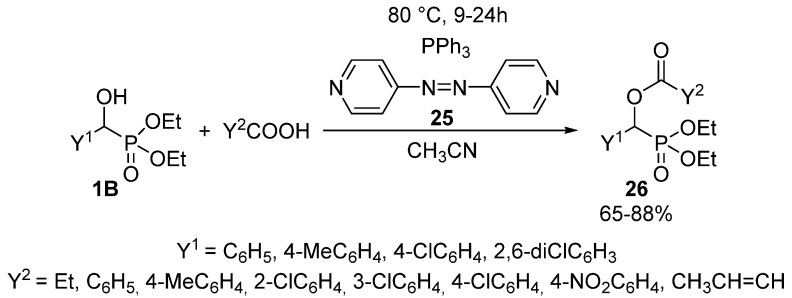
Synthesis of α-acyloxyphosphonates (**26**) via Mitsunobu reaction.

Another method of choice for the acylation of α-hydroxyphosphonates **1B** with carboxylic acids, is the Mitsunobu reaction applying 4,4′-azopyridine (**25**) and triphenylphosphine in acetonitrile as the solvent (Scheme 12) [92]. Completion of the reaction required a reaction time of 9–24 h at 80 °C, and the products **26** were obtained in yields of 65–88%.

**Scheme 13:**
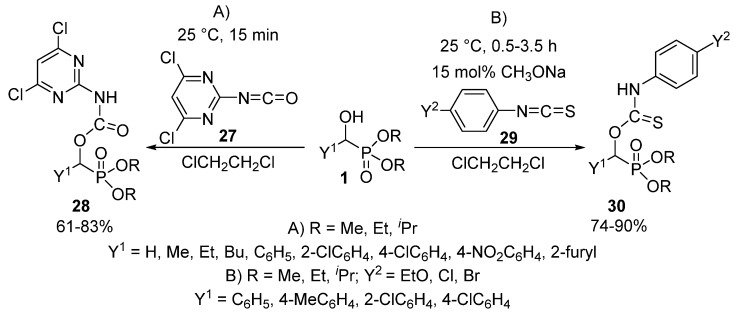
The reaction of α-hydroxyphosphonates (**1**) with isocyanates **27** and isothiocyanates **29**.

The reaction of α-hydroxyphosphonates **1** with isocyanates **27** or isothiocyanates **29** resulted in the formation of α-carbamoyloxy-**28** or α-thiocarbamoyloxyphosphonates **30**, respectively [93,94]. One study targeted herbicides by the acylation of α-hydroxyphosphonates (**1**) with pyrimidine-based isocyanates **27** (Scheme 13, Route “A”) [93]. 

The reaction took place under benign conditions, and the products **28** so-obtained revealed a weak herbicidal activity. On reacting hydroxyphosphonates (**1**) and isothiocyanates **29** in the absence of a catalyst, the corresponding α-thiocarbamoyl-oxyphosphonates **30** were obtained in modest yields of 20–30%. However, the application of 15 mol % of sodium methoxide promoted the acylation, and the products **30** could be isolated in yields of 74–90% (Scheme 13, Route “B”) [94]. The authors of the article emphasize the need for mild reaction conditions (25 °C, 0.5–3.5 h), as heating of the reaction mixture resulted in the formation of a by-product through the trimerization of the starting isothiocyanate. The α-thiocarbamoyl-oxyphosphonates **30** obtained in this way expressed plant growth regulating activity.

**Scheme 14:**
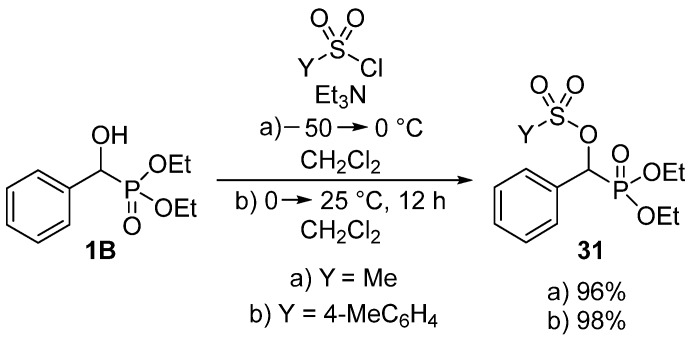
Sulfonylation of α-hydroxyphosphonates **1B**.

As shown above, the acylation of α-hydroxyphosphonates (**1B**) was carried out with a wide range of carboxylic acid derivatives. Besides carboxylic chlorides, anhydrides and acids, sulfonic acid derivatives may also function as the acylating agents. By analogy with the reaction of α-hydroxyphosphonates (**1B**) with carboxylic chlorides, the acylation was also attempted with sulfonyl chlorides [95,96,97]. Methanesulfonyl chloride (Scheme 14, Route “a”) [96] and *p*-toluenesulfonyl chloride (Scheme 14, Route “b”) [97] worked well as sulfonylating agents in the presence of triethylamine.

**Scheme 15:**
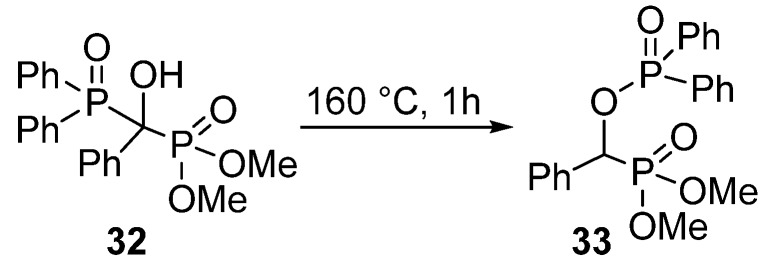
Synthesis of *O*-phosphoryloxy phosphonate **33** by rearrangement.

In contrast to the wide range of α-acyloxy and α-sulfonyloxy phosphonates, α-phosphoryloxy analogues appear rarely in the literature. A study from the early 1960’s reported the synthesis of a >P(O)OCHP(O)< type compound **33** by a thermo-induced rearrangement (Scheme 15) [98]. However, the phosphorylation of α-hydroxyphosphonates (**1**) with *P*-chlorides presented a challenge.

**Scheme 16:**
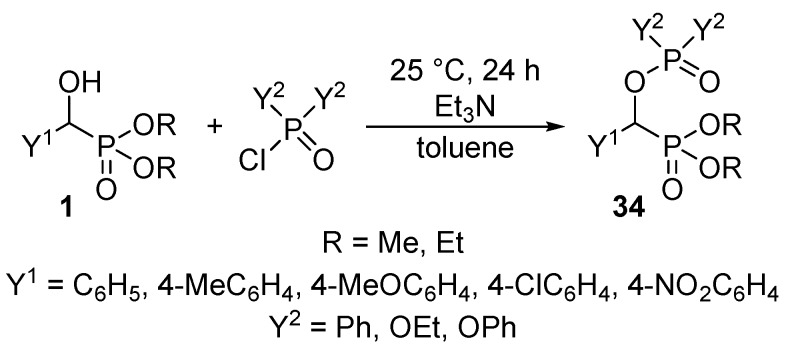
Phosphorylation of α-hydroxyphosphonates **1** with phosphoryl and phosphinic chlorides.

Recognizing the lack of *O*-phosphorylated α-hydroxyphosphonates **34** in the literature, we aimed at the synthesis of new classes of derivatives by the acylation of α-hydroxyphosphonates **1** with phosphoryl and phosphinic chlorides. It was observed that by stirring the reaction components at ambient temperature, the *O*-phosphorylated phosphonates **34** were formed with conversions of 55–90% (Scheme 16) [99].

### 2.3. Oxidations

**Scheme 17:**
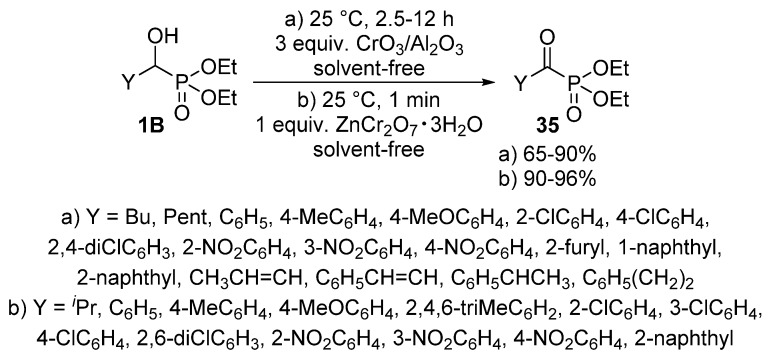
Oxidation of α-hydroxyphosphonates **1B** using metal compounds with variable valency.

The oxidation of α-hydroxyphosphonates **1B** affords the corresponding α-ketophosphonates (**35**) that are versatile precursors of a series of organophosphorus compounds [100,101,102]. The application of metal compounds with variable valency as the oxidizing agent is predominating in the literature. Alumina-supported chromium(III) oxide (CrO_3_/Al_2_O_3_) (Scheme 17, Route “a”) [103], and chromium salts, like zinc dichromate trihydrate (ZnCr_2_O_7_·3H_2_O) (Scheme 17, Route “b”) [104] were found to be efficient reagents in the oxidation of α-hydroxyphosphonates **1B**. In both cases, the reaction took place at ambient temperature under solvent-free conditions. The procedures provided the desired products **35** in good to excellent yields (up to 96%).

**Scheme 18:**
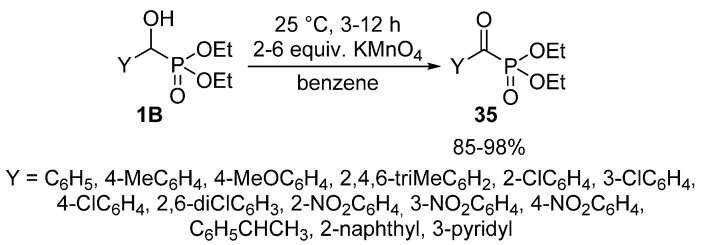
Synthesis of α-ketophosphonates **35** from α-hydroxyphosphonates **1B** with KMnO_4_ oxidant.

In other variations, pyridinium dichromate [105] or pyridinium chlorochromate [106] were applied successfully to give α-ketophosphonates **35**. The reaction under discussion was also reported using KMnO_4_ as the oxidant (Scheme 18). A shortcoming of this latter protocol is the need for dry benzene as the reaction medium [107]. Another problem of the chemical oxidating agents is the low atom efficiency and the lack of “greenness”.

**Scheme 19:**
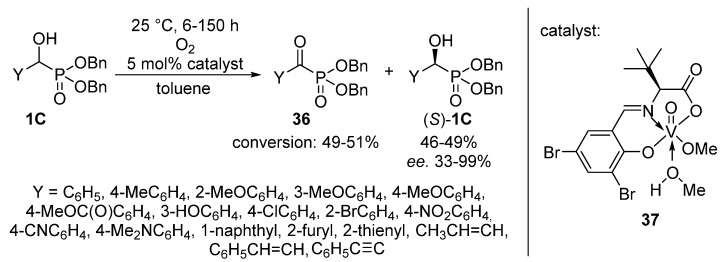
Tandem resolution and oxidation of α-hydroxyphosphonates **1C**.

An elegant achievement is the oxidation of dibenzyl α-hydroxyphosphonates **1C** with oxygen, employing a chiral vanadyl(V) complex (**37**) as the catalyst (Scheme 19) [108]. After a reaction time of 6–150 h, the corresponding α-ketophosphonates **36** were obtained in conversions of 49–51%, while due to the presence of the optically active catalyst, the (*S*) enantiomer of the α-hydroxyphosphonate (*S*)-**1C** remained untouched. This protocol offered an efficient way for the resolution of α-hydroxyphosphonates **1C** with high enantiomeric excesses up to 99% (with two exceptions), in parallel with the formation of α-ketophosphonates **36**.

### 2.4. Nucleophilic Substitutions

**Scheme 20:**
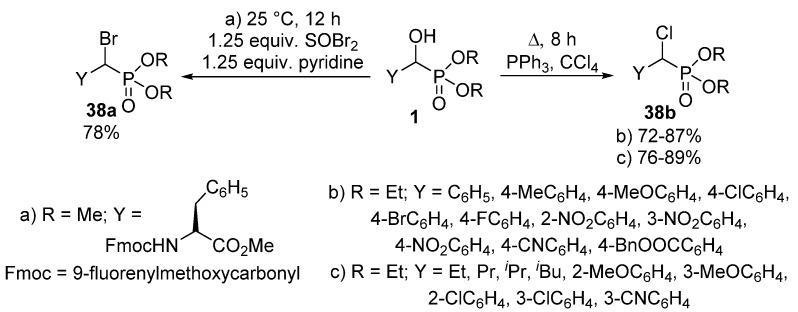
Synthetic routes to obtain α-halophosphonates **38**.

The nucleophilic substitution of the hydroxy group of α-hydroxyphosphonates **1** represents one of the most widely studied reaction types of this class of compounds. A typical transformation is the change of the hydroxy function to a halogen atom. α-Halobenzylphosphonates **38** deserve interest as potential inhibitors of protein tyrosine phosphatases [109,110]. A chloro or bromo substituent may be introduced into an α-hydroxyphosphonate **1** molecule by treatment with thionyl chloride [111] or thionyl bromide (Scheme 20, Route “a”) [110], respectively. Another method of choice to obtain α-halobenzylphosphonates **38** is the joint application of triphenylphosphine and the corresponding carbon tetrahalide [112,113,114]. One procedure applied this synthetic route to obtain α-chlorobenzylphosphonates (Scheme 20, Route “b”) [114], while another paper reported the introduction of a chloro atom to both aliphatic and aromatic hydroxyphosphonates under similar conditions (Scheme 20, Route “c”) [113].

A further variation involves the use of tetrabutylammonium bromide as the nucleophile in the presence of triphenylphosphine—DDQ (2,3-dichloro-5,6-dicyanobenzoquinone) system, affording α-bromophosphonates [115,116].

**Scheme 21:**
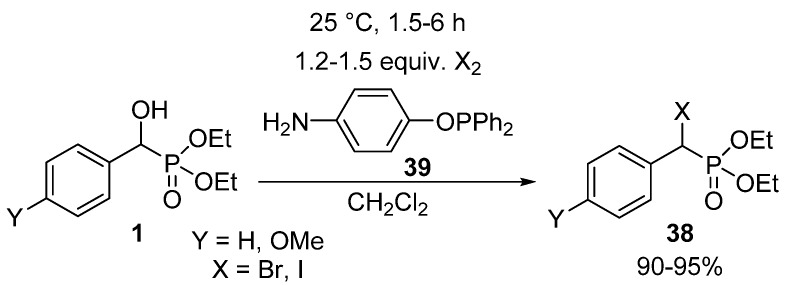
Synthesis of α-halophosphonates **38** from α-hydroxyphosphonates **1** with molecular halogens.

In another protocol, hydroxyphosphonates **1** were converted to their corresponding α-halo analogues **38** by reaction with molecular halogens (Br_2_ or I_2_) applying 4-aminophenyl-diphenylphosphinite **39** as the acid scavenger (Scheme 21) [117]. The amino function of the phosphinite **39** made possible the removal of the forming hydrogen halogenide as an ammonium salt after a simple filtration.

To synthesize α-fluorophosphonates, the most commonly used fluorinating agent is diethylaminosulfur trifluoride (Et_2_NSF_3_) [111,114,118,119,120,121]. As a variation of this method, a protocol applying morpholinosulfur trifluoride was elaborated for the OH → F change in α-hydroxy-bisphosphonates [122]. *N*-fluorobisbenzenesulfonimide was used efficiently to introduce a fluoro function to the α-carbon atom of α-monohalophosphonates **38** in the presence of sodium hexamethyldisilazane [113,114,116].

**Scheme 22:**
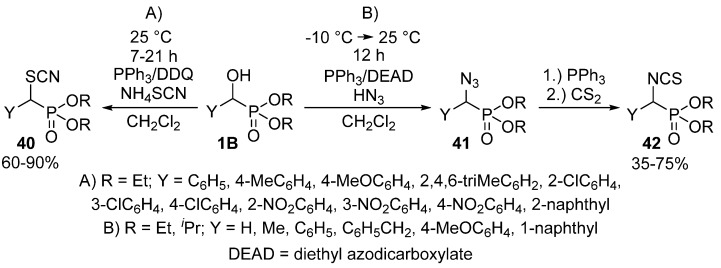
Nucleophilic substitution of α-hydroxyphosphonates **1B** with NH_4_SCN and hydrazoic acid.

Halogens are not the sole nucleophiles that the α-hydroxy function of benzylphosphonates **1B** may be replaced with. One method operated with the above mentioned triphenylphosphine−DDQ system to catalyze the reaction of α-hydroxyphosphonates **1B** with ammonium thiocyanate to obtain α-thiocyanatophosphonates **40** (Scheme 22, Route “A”) [115]. Another study reported the Mitsunobu reaction of α-hydroxyphosphonates **1B** with hydrazoic acid as the nucleophile. The treatment of the so-formed α-azidophosphonates **41** with triphenylphosphine and carbon disulfide resulted in the formation of α-isothiocyanatophosphonates **42** (Scheme 22, Route “B”) [123].

**Scheme 23:**
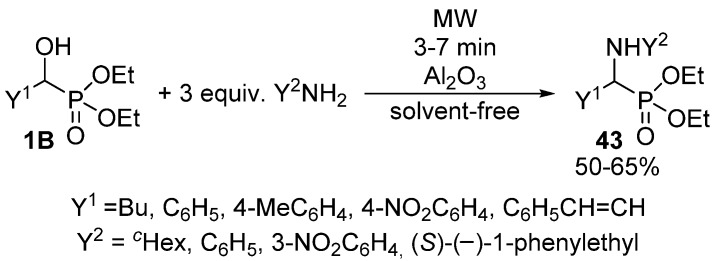
Synthesis of α-aminophosphonates **43** by nucleophilic substitution of **1B** with amines.

α-Aminophosphonates **43** are usually obtained by the three-component condensation (Kabachnik-Fields reaction) of oxo compounds, amines and dialkyl phosphites or secondary phosphine oxides [124,125,126,127]. However, these compounds may also be synthesized via the nucleophilic substitution of α-hydroxyphosphonates **1B** with primary or secondary amines. According to a simple protocol, this latter reaction was performed under MW irradiation, in the presence of Al_2_O_3_ [128]. After 3–7 min, a complete conversion was attained (Scheme 23). However, reaction temperatures are missing from the articles, as the experiments were carried out in a kitchen MW oven.

**Scheme 24:**
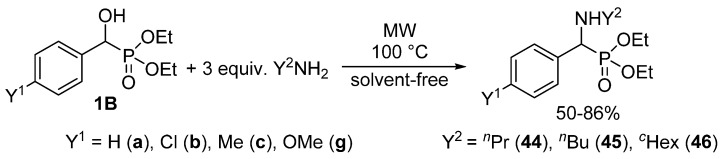
MW-Assisted synthesis of α-aminophosphonates **44**–**46** through nucleophilic substitution.

We found that α-hydroxyphosphonates **1B** may be converted to the corresponding α-aminophosphonates **44**–**46** under MW conditions without the use of any catalyst or solvent (Scheme 24) [56,129]. The starting hydroxyphosphonates **1B** were reacted with three equivalents of a primary amine (propyl-, butyl- or cyclohexyl-amine) applying MW irradiation at 100 °C. Following this method, the substitution took place with surprising ease. Starting from substituted hydroxyphosphonates **1Bb**, **1Bc** and **1Bg**, a short reaction time of 15–30 min was sufficient for complete conversion (Table 3/Entries 4–12). The unsubstituted analogue **1Ba** was even more reactive, and in this case the reaction took place after 10–15 min (Table 3/Entries 1–3).

**Scheme 25:**
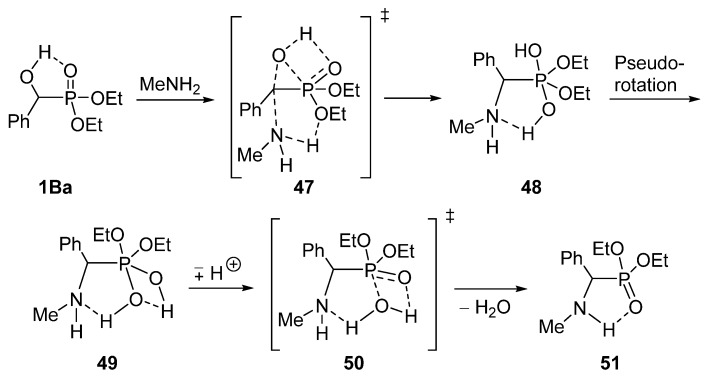
Calculated mechanism of the substitution of hydroxyphosphonate **1Ba** with methylamine.

Encouraged by the recognition that the reaction of α-hydroxyphosphonates **1B** with primary amines takes place rather easily, we wished to study the mechanism of the substitution by quantum chemical (DFT) calculations [129]. The nucleophilic attack of methylamine on diethyl α-hydroxybenzylphosphonate **1Ba** was chosen as the model reaction. It was found that the reaction follows an S_N_2 mechanism. It was also revealed that a favorable neighboring group effect facilitates the transfer of the oxygen atom of the hydroxy group from the α-carbon atom to the phosphorus (transition state **47**) [129]. The proposed mechanism is shown on Scheme 25.

α-Sulfonamidophosphonates were obtained through the reaction of α-hydroxyphosphonates and sulfonamides in the presence of HOTf in dioxane. The reaction took place at ambient temperature within 5 h, and the desired products were obtained in yields of 70–94% [130]. The products so-obtained were found to be as promising corrosion inhibitors for mild steel [131].

**Scheme 26:**
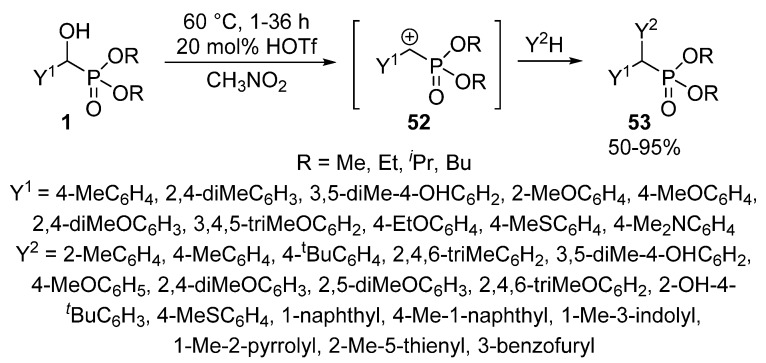
Friedel-Crafts alkylation of α-hydroxyphosphonates **1** with arene nucleophiles.

The Friedel-Crafts alkylation using α-hydroxyphosphonates **1** in the presence of an acid catalyst (e.g., FeCl_3_ or HOTf) led to α,α-diarylphosphonates **53** [132,133,134]. Benzene and naphthalene served as the aromatic substrate. According to the proposed mechanism, the first step is the formation of benzylphosphonate carbocation **52** that is followed by its electrophilic attack on the aromatic substrate (Scheme 26) [132].

**Scheme 27:**
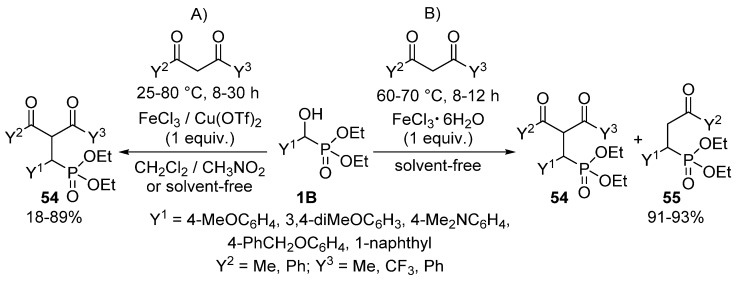
Alkylation of 1,3-diketones with α-hydroxyphosphonates **1B**.

An interesting reaction is when α-hydroxyphosphonates **1B** function as an alkylating agent of 1,3-diketones to afford γ-ketophosphonates **54** (Scheme 27) [135]. The reaction of the starting components in organic solvents in the presence of FeCl_3_ or Cu(OTf)_2_ Lewis acids resulted in the formation of compound **54** (Scheme 27, Route “A”). The optimization of the alkylations was a real challenge for the authors, as each hydroxyphosphonate—diketone combination required different reaction conditions regarding the Lewis acid, temperature, time and solvent. However, applying FeCl_3_·6H_2_O as the catalyst, a *C*–*C* bond cleavage of the desired product **54** also occurred due to the presence of the crystal hydrate of the iron salt. In this case, a mixture of compounds **54** and **55** was attained (Scheme 27, Route “B”).

### 2.5. Rearrangements

The rearrangement of α-hydroxyphosphonates **1** to benzyl phosphates was first discovered through the example of the widely known insecticide prodrug, trichlorofon (*O*,*O*-dimethyl (2,2,2-trichloro-1-hydroxyethyl)phosphonate) that is rearranged to 2,2-dichlorovinyl dimethyl phosphate (DDVP) acting as an acetylcholinesterase inhibitor [136]. Phosphonate-phosphate rearrangements were investigated in the presence of strong bases, such as sodium hydroxide [136], sodium ethoxide [137] and sodium hydride [138]. A number of protocols operated with triethylamine as the catalyst [139,140,141]. However, the Et_3_N-catalyzed rearrangement is not a versatile method, as it was reported to apply to only six-membered cyclic phosphonates [139,140,141].

**Scheme 28:**
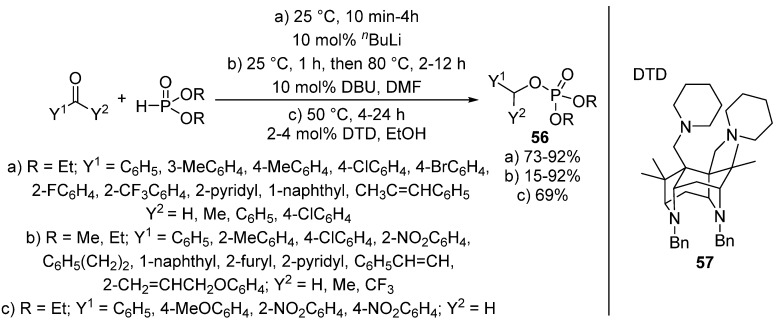
Methods of the tandem Pudovik reaction and rearrangement affording benzyl phosphates **56**.

A new approach of this transformation is when the Pudovik reaction of benzaldehydes and dialkyl phosphites and the subsequent rearrangement of the so-formed α-hydroxyphosphonate **1** take place in “one-pot”, under the same conditions. The synthesis of benzyl phosphates **56** directly from an oxo compound and a *P*-reagent was efficiently catalyzed by butyllithium (Scheme 28, Route “a”) [142] as well as by DBU (Scheme 28, Route “b”) [143]. The tandem Pudovik reaction and rearrangement was also reported in the presence of superbases **57** (Scheme 28, Route “c”) [144]. The authors of this latter article emphasized that both the reaction media and the substituent in the aromatic ring had a great impact on the reaction. The rearrangement took place faster in alcohols (EtOH or *^i^*PrOH) than in acetonitrile. It was also shown that the presence of electron-withdrawing substituents facilitated the formation of the benzyl phosphate **56** [144].

**Scheme 29:**
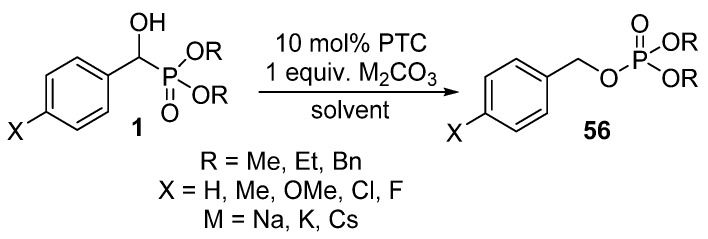
Rearrangement of α-hydroxyphosphonates **1** under phase-transfer catalytic conditions.

In this field, our research targets the accomplishment of the rearrangement of α-hydroxyphosphonates **1** using bases under phase transfer catalytic conditions (Scheme 29) [145].

Our preliminary results highlighted that the substituent in the aromatic ring has a significant impact on the reaction. In the presence of electron-withdrawing substituents (Cl and F), the reaction took place at ambient temperature, whereas the rearrangement of α-hydroxyphosphonates bearing electron-releasing substituents required 2–3 h of heating [145].

### 2.6. Hydrolysis

The hydrolysis of α-hydroxyphosphonates **1** is the most common way for the synthesis of α-hydroxyphosphonic acids **12** that may be inhibitors of a wide variety of enzymes including protein tyrosine phosphatases [110,146,147], mandelate racemase [148], phosphoglycerate kinase [149], plant P5C reductase [150] and plant glutamine synthetase [151]. A series of α-hydroxyphosphonic acid (**12**) and α-hydroxybisphosphonic acid was recognized as antibiotics against Gram-positive bacteria [152]. α-Hydroxyphosphonic acids **12** bearing an amide function in the *para*-position of the aromatic ring were found to be in vivo inhibitors of autotaxin [153].

As for the synthesis of α-hydroxyphosphonic acids **12**, most of the related articles reported the acidic hydrolysis of the corresponding methyl or ethyl ester [149,150,152,154]. A number of protocols applied hydrochloric acid in an excess at reflux, and the hydrolysis usually required a prolonged reaction time of 6–24 h [149,150,152]. According to another method, beside the excess of 6 M hydrochloric acid, dioxane was also added as a co-solvent [154].

**Scheme 30:**
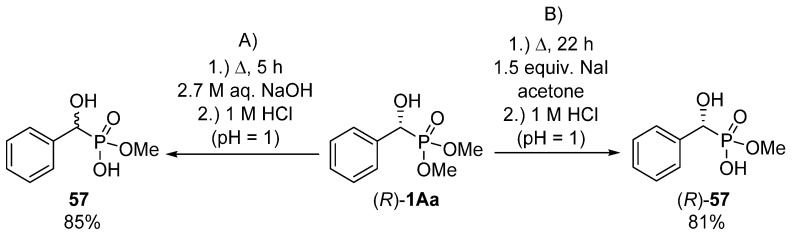
Selective hydrolysis of one ester function of hydroxyphosphonate (*R*)-**1Aa**.

In the hope of selective hydrolysis of one of the ester functions, the hydrolytic reaction of dimethyl 1-hydroxy-1-phenylmethylphosphonate **1Aa** was carried out in aqueous sodium hydroxide at reflux for 5 h (Scheme 30, Route “A”) [155]. After pH adjustment, the desired product (**57**) was obtained in a yield of 85%. This method was suitable only for the synthesis of the racemic target compound **57**. In order to maintain the *C* chirality center of the starting α-hydroxyphosphonate (*R*)-**1Aa**, a milder protocol had to be elaborated. On stirring the starting optically active α-hydroxyphosphonate (*R*)-**1Aa** in the presence of sodium iodide in acetone at reflux for 22 h, racemization of the hydroxyphosphonate (*R*)-**1Aa** could be avoided (Scheme 30, Route “B”) [155].

Another way of synthesizing α-hydroxyphosphonic acids (**12**) from α-hydroxyphosphonates **1** involves the cleavage of the C–O bond of the ester function by trimethylsilyl chloride [77,138] or trimethylsilyl bromide [110]. This reaction is usually carried out at ambient temperature within 2–18 h, generally in acetonitrile as the solvent.

## 3. Conclusions

This review summarizes the synthesis and reactions of α-hydroxyphosphonates that are of importance due to their bioactivity. The first half of the review presents the most common synthetic routes towards α-hydroxyphosphonates, with special stress on the addition of dialkyl phosphite to an oxo compound, and the condensation of trialkyl phosphites with aldehydes or ketones. The overview is followed by the discussion of the green synthetic protocols for α-hydroxyphosphonates. Then, the reactions of α-hydroxyphosphonates involving *O*-alkylation, *O*-acylation and oxidation of the hydroxy function, substitutions of the hydroxy group by a chloro or amino function, rearrangements to phosphate derivatives and hydrolysis of the ester function are discussed. Beside the literature examples, our own synthetic results are also included.

## Figures and Tables

**Table 1:**
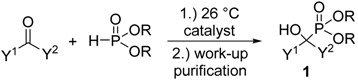
Selected examples for the synthesis of α-hydroxyphosphonates (**1**) from aldehydes or ketones and dialkyl phosphites (method “A”).

Entry	Y^1^	Y^2^	R	Catalyst	Amount of Catalyst	Conditions	Yield (%)	Ref.
1	*^i^*Pr, *^c^*Hex, C_6_H_5_, 4-MeC_6_H_4_, 4-*^i^*PrC_6_H_4_, 4-MeOC_6_H_4_, 3,4-diMeOC_6_H_3_, 4-ClC_6_H_4_, 2-NO_2_C_6_H_4_, 4-CNC_6_H_4_, C_6_H_5_CH=CH, 3,4-OCH_2_OC_6_H_3_, 4-CH_2_=CHCH_2_OC_6_H_4_, 4-PhCH_2_OC_6_H_4_, 2-furyl, 2-thienyl	H	Me, Et, *^i^*Pr	K_3_PO_4_	5 mol %	25 °C, 4–8 min for aromatic aldehydes, 24 h for aliphatic aldehydes	20–98	[21]
2	C_6_H_5_, 4-MeOC_6_H_4_, 3,4-diMeOC_6_H_3_, 2-ClC_6_H_4_, 4-ClC_6_H_4_, 2,3-diClC_6_H_3_, 2,4-diClC_6_H_3_, 2,6-diClC_6_H_3_, 2-BrC_6_H_4_, 4-BrC_6_H_4_, 3-FC_6_H_4_, 4-FC_6_H_4_, 2-NO_2_C_6_H_4_, 3-NO_2_C_6_H_4_, 4-NO_2_C_6_H_4_, 4-CNC_6_H_4_, C_6_H_5_CH=CH, 2-thienyl, 4-pyridyl	H	Et	Ba(OH)_2_	10 mol %	25 °C, 4–10 min	70–98	[22]
3	*^i^*Bu, C_6_H_5_, 2-MeC_6_H_4_, 3-MeC_6_H_4_, 4-MeC_6_H_4_, 4-EtC_6_H_4_, 4-MeOC_6_H_4_, 2,3,4-triMeOC_6_H_2_, 2-ClC_6_H_4_, 4-ClC_6_H_4_, 2,6-diClC_6_H_3_, 3-BrC_6_H_4_, 4-BrC_6_H_4_, 3-FC_6_H_4_, 4-FC_6_H_4_, 2-NO_2_C_6_H_4_, 3-NO_2_C_6_H_4_, 4-NO_2_C_6_H_4_, 3-CNC_6_H_4_, 4-CNC_6_H_4_, 2-furyl, 2-thienyl, 2-pyridyl, 3-pyridyl, 4-pyridyl, 1-naphthyl, 2-naphthyl, 9-anthryl, C_6_H_5_CH=CH	H	Et, Bu, Bn	Ba(OH)_2_·8H_2_O	2–7 mol %	25 °C, 15 min, THF	72–99	[23]
4	C_6_H_5_, 4-MeC_6_H_4_, 3-MeOC_6_H_4_, 4-MeOC_6_H_4_, 3-HOC_6_H_4_, 2-ClC_6_H_4_, 4-ClC_6_H_4_, 2,6-diClC_6_H_3_, 2-NO_2_C_6_H_4_, 3-NO_2_C_6_H_4_, 4-NO_2_C_6_H_4_, 4-Me_2_NC_6_H_4_, C_6_H_5_CH=CH, 2-furyl, 1-naphthyl, 2-naphthyl	H	Et	MgO	1 equiv.	25 °C, 2 min–4 h	80–100	[24]
5 *	C_6_H_5_, 4-MeC_6_H_4_, 4-MeOC_6_H_4_	(CH_2_)_2_CN, MeCHCH_2_CN	Me, Et	MgO	2 equiv.	25 °C, 1–6 h	70–82	[25]
6 *	Pr, Bu, C_6_H_5_, 4-MeC_6_H_4_, 4-MeOC_6_H_4_, 4-*^i^*PrC_6_H_4_, 2,4,6-triMeC_6_H_2_, 2-OHC_6_H_4_, 4-ClC_6_H_4_, 4-BrC_6_H_4_, 3-FC_6_H_4_, 4-FC_6_H_4_, 4-Br-2-OHC_6_H_3_, 3,4-diMeOC_6_H_3_, 3,4-OCH_2_OC_6_H_3_, 2-NO_2_C_6_H_4_, 4-NO_2_C_6_H_4_, 2-furyl, 2-thienyl, 3-thienyl, 1-naphthyl, 2-naphthyl	H, Me	Me	Al_2_O_3_	3 equiv.	25 °C, 72 h	52–98	[26]
7	C_6_H_5_, 4-MeC_6_H_4_, 4-MeOC_6_H_4_, 4-ClC_6_H_4_, 4-FC_6_H_4_, 4-NO_2_C_6_H_4_	H	Me, Et	Al_2_O_3_ + KF	1 + 2 equiv.	25 °C, 30 min	53–88	[27]
8 *	Me, ClCH_2_, C_6_H_5_, 4-MeC_6_H_4_, 4-MeOC_6_H_4_, 2-ClC_6_H_4_, 3-ClC_6_H_4_, 4-ClC_6_H_4_, 3-BrC_6_H_4_, 4-BrC_6_H_4_, 2-FC_6_H_4_, 4-FC_6_H_4_, 2,4-diClC_6_H_3_, 2-furyl, 2-thienyl	Me, Pr, Ph	Me	Et_3_N	1 equiv.	40 °C, 2 h	63–89	[28]
9 *	Pr, C_6_H_5_, 4-MeC_6_H_4_, 4-MeOC_6_H_4_, 2-ClC_6_H_4_, 4-ClC_6_H_4_, 3-NO_2_C_6_H_4_, 4-NO_2_C_6_H_4_, 4-CNC_6_H_4_, (CH_2_)_2_C_6_H_5_, C_6_H_5_CH=CH, 2-furyl, 2-naphthyl, 9-anthryl	H, Me	Me	Et_3_N + MgCl_2_	3 + 1 equiv.	50 °C, 1–2 h	85–98	[29]
10	C_6_H_5_, 4-MeC_6_H_4_, 4-MeOC_6_H_4_, 4-OHC_6_H_4_, 2-ClC_6_H_4_, 4-ClC_6_H_4_, 4-BrC_6_H_4_, 2-NO_2_C_6_H_4_, 3-NO_2_C_6_H_4_, 4-NO_2_C_6_H_4_	H	Et	–	–	MW, 90–100 °C, 10–20 min	79–95	[30]
11	C_6_H_5_, 4-MeC_6_H_4_, 4-MeOC_6_H_4_, 4-ClC_6_H_4_, 4-NO_2_C_6_H_4_	H	Me, Et	Na_2_CO_3_	0.75 equiv.	MW, 110 °C, 20 min	62–88	[31]
12	C_6_H_5_, 4-MeC_6_H_4_, 4-MeOC_6_H_4_, 4-ClC_6_H_4_, 3-NO_2_C_6_H_4_, 4-NO_2_C_6_H_4_	H	Et	Na_2_CO_3_	1 equiv.	Grinding, 25 °C, 10 min	75–83	[32]
13	C_6_H_5_, 4-MeC_6_H_4_, 4-*^i^*PrC_6_H_4_, 4-MeOC_6_H_4_, 3,4-diMeOC_6_H_3_, 2-ClC_6_H_4_, 4-ClC_6_H_4_, 2-NO_2_C_6_H_4_, 3-NO_2_C_6_H_4_, 4-NO_2_C_6_H_4_, 4-Me_2_NC_6_H_4_, 4-PhCH_2_OC_6_H_4_, 4-C_5_H_4_NC_6_H_4_, 9-anthryl	H	Et	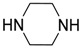	1 equiv.	Grinding, 25 °C, 2–10 min	78–96	[33]
14	C_6_H_5_, 2-MeC_6_H_4_, 4-MeC_6_H_4_, 2-*^i^*PrC_6_H_4_, 3,5-diMeC_6_H_3_, 2-MeOC_6_H_4_, 4-MeOC_6_H_4_, 3,5-diMeOC_6_H_3_, 3,4,5-triMeOC_6_H_2_, 4-EtOC_6_H_4_, 2-ClC_6_H_4_, 2-BrC_6_H_4_, 3-BrC_6_H_4_, 4-BrC_6_H_4_, 2-Cl-6-furylC_6_H_3_, 2-F-4-BrC_6_H_3_, 2-NO_2_C_6_H_4_, 4-NO_2_C_6_H_4_, 9-anthryl, 1-pyrenyl, 2-furyl, 1-C_6_H_5_-4-pyrazolyl	H	Et	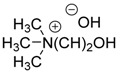	10 mol %	25 °C, 5–10 min	90–98	[34]
15 *	C_6_H_5_, 3-MeOC_6_H_4_, 4-MeOC_6_H_4_, 4-ClC_6_H_4_, 3-NO_2_C_6_H_4_, 4-NO_2_C_6_H_4_, C_6_H_5_CH=CH	H, Me	Me, Et	Na-modified fluoroapatite	1 g/1.25 mol acetophenone	20–25 °C, 1–1.5 min	75–98	[35]
16	4-*^i^*PrC_6_H_4_, 4-MeOC_6_H_4_, 2,6-diMeOC_6_H_3_, 3,5-diMeOC_6_H_3_, 4-OHC_6_H_4_, 3-NO_2_C_6_H_4_, 4-Me_2_NC_6_H_4_, 2-PhCH_2_OC_6_H_4_, 2-imidazyl, 3-indolyl	H	Et	KHSO_4_	20 mol %	25 °C, 2–4 h	82–91	[36]
17	3-FC_6_H_4_, 4-NO_2_C_6_H_4_, 3,4-OCH_2_OC_6_H_3_, 4-MeSC_6_H_4_, C_5_H_10_N, 3-MeO-4-OHC_6_H_3_, 4-C_4_H_8_NC_6_H_4_, 2-furyl, 2-thienyl, 4-imidazyl, 2-pyrrolyl, 4-pyridyl	H	Me	silica-supported tungstic acid	20 mol %	25 °C, 30 min	85–96	[37]
18 *	Me, Pr, *^i^*Bu, Pent, C_6_H_5_, 2-MeC_6_H_4_, 4-MeC_6_H_4_, 2-MeOC_6_H_4_, 3-MeOC_6_H_4_, 4-MeOC_6_H_4_, 2-ClC_6_H_4_, 3-ClC_6_H_4_, 4-ClC_6_H_4_, 2-BrC_6_H_4_, 4-BrC_6_H_4_, 4-FC_6_H_4_, 3-NO_2_C_6_H_4_, 4-NO_2_C_6_H_4_, 1-naphthyl, 2-naphthyl, 2-furyl, 2-thienyl, 3-pyridyl	H, Me, Ph, CF_3_, (CH_2_)_10_CH_3,_ C(O)Ph, CH_2_C(O)Ph	Et, *^i^*Pr, Ph	BuLi	0.1 mol %	10–25 °C, 5 min, hexane	35–99	[38]
19 *	Et, *^c^*Hex, C_6_H_5_, 3-MeC_6_H_4_, 4-MeC_6_H_4_, 3-MeOC_6_H_4_, 4-ClC_6_H_4_, 3,4-diClC_6_H_3_, 2,3,4-triClC_6_H_2_, 2-FC_6_H_4_, 4-FC_6_H_4_, 4-NO_2_C_6_H_4_, 3-CF_3_C_6_H_4_CH2=CH, 3-CF_3_C_6_H_4_CH(OH)CH_2_, 2-thienyl, 2-naphthyl, EtOC(O)CHBn	Me, Et, Ph, CH(OEt)_2_, COOMe, (CH_2_)_2_Cl	Me	Ti(O*^i^*Pr)_4_	5 mol %	30 °C, 15 min	74–98	[39]
20	C_6_H_5_, 4-MeOC_6_H_4_, 4-FC_6_H_4_, 4-NO_2_C_6_H_4_, 4-CF_3_C_6_H_4_, 4-CNC_6_H_4_, 4-MeOC(O)C_6_H_4_, (CH_2_)_2_C_6_H_5_, C_6_H_5_CH=CH	H	Et	MoO_2_Cl_2_	5 mol %	80 °C, 1–24 h	70–96	[40]

* The procedure is also suitable starting from ketones.

**Table 2:**
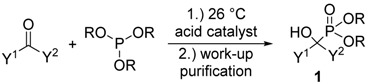
Selected procedures to synthesize α-hydroxyphosphonates (**1**) from aldehydes or ketones and trialkyl phosphites (method “B”).

Entry	Y^1^	Y^2^	R	Catalyst	Amount of Catalyst	Conditions	Yield (%)	Ref.
1 *	C_6_H_5_, 4-MeC_6_H_4_, 4-*^i^*PrC_6_H_4_, 4-MeOC_6_H_4_, 2-ClC_6_H_4_, 4-ClC_6_H_4_, 4-BrC_6_H_4_, 4-FC_6_H_4_, 4-NO_2_C_6_H_4_, 4-CF_3_C_6_H_4_, 4-Me_2_NC_6_H_4_, CH_2_C_6_H_5_, (CH_2_)_2_C_6_H_5_	H, Me, Et, Ph	Me, Et	–	–	Sonication, 25 °C, 10–35 min	84–94	[41]
2	Pr, *^i^*Bu, C_6_H_5_, 4-MeC_6_H_4_, 4-MeOC_6_H_4_, 3-OHC_6_H_4_, 2-ClC_6_H_4_, 4-ClC_6_H_4_, C_6_H_5_CH=CH, 3-chromonyl, 6-Cl-7-Me-3-chromonyl, 2-Cl-3-quinolinyl	H	Et	KH_2_PO_4_	5 mol %	Sonication, 25 °C, 5–45 min	48–92	[42]
3	C_6_H_5_, 4-MeC_6_H_4_, 4-MeOC_6_H_4_, 4-ClC_6_H_4_, 3-pyridyl, 3-chromonyl, 6,8-diMe-3-chromonyl, 2-Cl-3-quinolinyl, 6-Cl-3-chromonyl, 6,7-diCl-3-chromonyl, 6,8-diCl-3-chromonyl, 6-Br-3-chromonyl, 6-Cl-7-Me-3-chromonyl, 2-Cl-6-Me-3-quinolinyl, 2-Cl-7-Me-3-quinolinyl, 2-Cl-8-Me-3-quinolinyl, 2-Cl-6-MeO-3-quinolinyl, 2-Cl-6-EtO-3-quinolinyl, 2-Cl-8-Et-3-quinolinyl	H	Me, Et	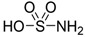	25 mol %	Sonication, 25 °C, 1–60 min	78–98	[43]
4	C_6_H_5_, 4-MeC_6_H_4_, 4-MeOC_6_H_4_, 4-OHC_6_H_4_, 4-ClC_6_H_4_, 4-NO_2_C_6_H_4_, 3,4-OCH_2_OC_6_H_3_, C_6_H_5_CH=CH, 2-furyl, 2-thienyl, 2-Cl-3-quinolinyl, 4-tetrazolo[1,5-*a*]quinolinyl	H	Et	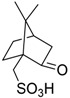	10 mol %	Sonication, 25 °C, 8–20 min	85–93	[44]
5	Pr, *^i^*Pr, Pent, *^c^*Hex, C_6_H_5_, 4-MeC_6_H_4_, 4-MeOC_6_H_4_, 4-OHC_6_H_4_, 2-ClC_6_H_4_, 4-ClC_6_H_4_, 4-CNC_6_H_4_, C_6_H_5_CH=CH, 2-furyl	H	Me	(COOH)_2_	10 mol %	80 °C, 3 h	83–98	[45]
6	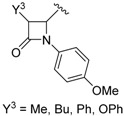	H	Me, Et	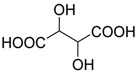	10 mol %	∆, 30 min, acetonitrile	41–69	[46]
7 *	Pr, C_6_H_5_, 4-MeC_6_H_4_, 4-MeOC_6_H_4_, 2-ClC_6_H_4_, 4-ClC_6_H_4_, 3-NO_2_C_6_H_4_, 4-NO_2_C_6_H_4_, 4-CNC_6_H_4_, (CH_2_)_2_C_6_H_5_, C_6_H_5_CH=CH, 2-furyl	H, Me	Me	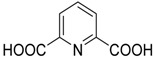	10 mol %	50 °C, 1.3–3 h, H_2_O	60–95	[47]
8	Pr, *^i^*Pr, Bu, *^t^*Bu, Hex, *^c^*Hex, C_6_H_5_, 4-ClC_6_H_4_, 2-furyl, 2-pyridyl	H	Me	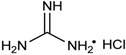	0.5 mol %	50 °C, 2 h, H_2_O	60–95	[48]
9	Me, Et, *^i^*Pr, C_6_H_5_, 4-MeC_6_H_4_, 4-MeOC_6_H_4_, 2-ClC_6_H_4_, 4-ClC_6_H_4_, 2,4-diClC_6_H_3_, 2-NO_2_C_6_H_4_, 3-NO_2_C_6_H_4_, 4-NO_2_C_6_H_4_, C_6_H_5_CH=CH, 2-furyl, 2-thienyl	H	Et	I_2_	10 mol %	80 °C, 15–120 min, H_2_O	83–97	[49]
10	C_6_H_5_, 4-MeC_6_H_4_, 4-MeOC_6_H_4_, 2-OHC_6_H_4_, 4-ClC_6_H_4_, 4-BrC_6_H_4_, 4-FC_6_H_4_, 3-NO_2_C_6_H_4_, 4-NO_2_C_6_H_4_, 2-pyridyl, 2-naphthyl, 2-furyl, 2-thienyl	H	Et	β-cyclodextrin	1 equiv.	60–70 °C, 8–12 h, H_2_O	80–93	[50]
11	C_6_H_5_, 4-MeC_6_H_4_, 4-MeOC_6_H_4_, 3-OHC_6_H_4_, 2-ClC_6_H_4_, 4-ClC_6_H_4_, 4-NO_2_C_6_H_4_, C_6_H_5_CH=CH, 3-chromonyl, 6-Cl-3-chromonyl, 6,7-diCl-3-chromonyl, 6,8-diCl-3-chromonyl, 6-Cl-7-Me-3-chromonyl, 2-Cl-3-quinolinyl, 2-Cl-6-Me-3-quinolinyl, 2-Cl-7-MeO-3-quinolinyl, 2-Cl-8-Et-3-quinolinyl, 2-Cl-6-EtO-3-quinolinyl	H	Et	NH_4_VO_3_	10 mol %	25 °C, 5–40 min	80–94	[51]
12	4-ClC_6_H_4_, 2,4-diClC_6_H_3_, 4-BrC_6_H_4_, 4-MeOC(O)C_6_H_4_, 4-CF_3_C_6_H_4_, 2-NO_2_-3,6-diMeOC_6_H_2_, 1-naphthyl	H	Et	ZnBr_2_	10 mol %	25 °C, 10–30 min	68–91	[52]
13	C_6_H_5_, 4-MeC_6_H_4_, 4-MeOC_6_H_4_, 4-HOC_6_H_4_, 2-ClC_6_H_4_, 4-ClC_6_H_4_, 4-NO_2_C_6_H_4_, 4-Me_2_NC_6_H_4_, C_6_H_5_CH=CH, 2-Cl-3-quinolinyl, 2-Cl-6-Me-3-quinolinyl	H	Et	Bi(NO_3_)_3_·5H_2_O	10 mol %	MW, 70 °C, 10–15 min	88–95	[53]
14 *	Et, Pr, *^i^*Pr, *^t^*Bu, *^c^*Hex, CH_3_CH=CH, C_6_H_5_, 4-MeOC_6_H_4_, 4-ClC_6_H_4_, (CH_2_)_2_C_6_H_5_, C_6_H_5_CH=CH	H, Me	Me, Et	NbCl_5_, TMSCl	0.05 mol %	25 °C, 20–90 min	44–96	[54]

* The procedure is also suitable starting from ketones.

**Table 3:** Details of the synthesis of α-aminophosphonates **44**–**46** from α-hydroxyphosphonates **1B**.

Entry	Y^1^	Y^2^	Reaction Time (min)	Yield (%)	Product	Ref.
1	H	*^n^*Pr	10	78	**44a**	[129]
2	H	*^n^*Bu	15	86	**45a**	[129]
3	H	*^c^*Hex	10	84	**46a**	[129]
4	Cl	*^n^*Pr	15	60	**44b**	[50]
5	Cl	*^n^*Bu	20	50	**45b**	[56]
6	Cl	*^c^*Hex	30	54	**46b**	[56]
7	Me	*^n^*Pr	15	58	**44c**	[56]
8	Me	*^n^*Bu	15	79	**45c**	[56]
9	Me	*^c^*Hex	30	73	**46c**	[56]
10	OMe	*^n^*Pr	15	72	**44g**	[56]
11	OMe	*^n^*Bu	30	66	**45g**	[56]
12	OMe	*^c^*Hex	30	70	**46g**	[56]
